# Wnt7a Decreases Brain Endothelial Barrier Function Via β-Catenin Activation

**DOI:** 10.1007/s12035-023-03872-0

**Published:** 2023-12-26

**Authors:** Narek Manukjan, Steven Chau, Florian Caiment, Marcel van Herwijnen, Hubert J. Smeets, Daniel Fulton, Zubair Ahmed, W. Matthijs Blankesteijn, Sébastien Foulquier

**Affiliations:** 1 Department of Pharmacology and Toxicology, Maastricht University, 50 Universiteitssingel, P.O. Box 616, Maastricht, 6200 MD The Netherlands; 2 CARIM—School for Cardiovascular Diseases, Maastricht University, P.O. Box 616, Maastricht, 6200 MD The Netherlands; 3 Neuroscience and Ophthalmology, Institute of Inflammation and Ageing, University of Birmingham, Edgbaston, B15 2TT Birmingham UK; 4 Department of Toxicogenomics, GROW – School for Oncology and Developmental Biology, Maastricht University, P.O. Box 616, Maastricht, 6200 MD The Netherlands; 5 MHeNs—School for Mental Health and Neuroscience, Maastricht University, P.O. Box 616, Maastricht, 6200 MD The Netherlands; 6 Centre for Trauma Sciences Research, University of Birmingham, Edgbaston, Birmingham, B15 2TT UK; 7 Department of Neurology, Maastricht University Medical Center+, P.O. Box 5800, Maastricht, 6202 AZ The Netherlands

**Keywords:** Beta-catenin, TEER, BBB, Hypoxia, Vascular Dementia, cSVD

## Abstract

The blood-brain barrier consists of tightly connected endothelial cells protecting the brain’s microenvironment from the periphery. These endothelial cells are characterized by specific tight junction proteins such as Claudin-5 and Occludin, forming the endothelial barrier. Disrupting these cells might lead to blood-brain barrier dysfunction. The Wnt/β-catenin signaling pathway can regulate the expression of these tight junction proteins and subsequent barrier permeability. The aim of this study was to investigate the in vitro effects of Wnt7a mediated β-catenin signaling on endothelial barrier integrity. Mouse brain endothelial cells, bEnd.3, were treated with recombinant Wnt7a protein or XAV939, a selective inhibitor of Wnt/β-catenin mediated transcription to modulate the Wnt signaling pathway. The involvement of Wnt/HIF1α signaling was investigated by inhibiting Hif1α signaling with *Hif1α* siRNA. Wnt7a stimulation led to activation and nuclear translocation of β-catenin, which was inhibited by XAV939. Wnt7a stimulation decreased Claudin-5 expression mediated by β-catenin and decreased endothelial barrier formation. Wnt7a increased *Hif1α* and *Vegfa* expression mediated by β-catenin. However, *Hif1α* signaling pathway did not regulate tight junction proteins Claudin-5 and Occludin. Our data suggest that Wnt7a stimulation leads to a decrease in tight junction proteins mediated by the nuclear translocation of β-catenin, which hampers proper endothelial barrier formation. This process might be crucial in initiating endothelial cell proliferation and angiogenesis. Although HIF1α did not modulate the expression of tight junction proteins, it might play a role in brain angiogenesis and underlie pathogenic mechanisms in Wnt/HIF1α signaling in diseases such as cerebral small vessel disease.

**Supplementary Information:**

The online version contains supplementary material available at 10.1007/s12035-023-03872-0.

## Introduction

The blood-brain barrier (BBB) constitutes a highly specialized vascular structure, which separates the blood circulation from the central nervous system (CNS) and functions to control the passage of molecules and ions to the brain in a protective manner. The BBB prevents the entry of harmful toxins, inflammatory cells, and pathogens, while still providing oxygen and nutrients necessary for the normal functioning of the brain [1]. An important feature of the BBB is the specific characteristics of the endothelial cells (ECs) that line the blood vessels in the CNS, such as tight junctions (TJs) and reduced pinocytosis [2]. Other cells, such as astrocytes, pericytes and oligodendrocytes, are also involved in the tight regulation of the brain microenvironment, forming the neurovascular unit (NVU) [3].

Brain ECs possess TJ proteins and maintain a homeostatic environment by tightly holding cells together and thus form a protective structural barrier [4]. These TJ proteins also link adjacent brain ECs together by forming homodimer transmembrane proteins, and normal function of TJ proteins ensures the correct regulation of intercellular communication and paracellular transport [4, 5]. Claudins, one class of TJ proteins, exhibit homophilic binding to other claudins, and heterophilic binding to other TJ-associated proteins to form multiprotein junctional complexes between adjacent cells [6]. Claudin-5 (CLDN5) is the most abundant isoform in the BBB and is crucial for the regulation of its properties [5–8]. Dysfunction of ECs can disrupt TJ proteins and BBB function, ultimately leading to neurodegenerative disorders such as Alzheimer’s disease, multiple sclerosis, stroke, and cerebral small vessel disease (cSVD) [4, 8–10]. Dysregulation of secreted factors, such as Wingless-related integration site (Wnt), by cells of the NVU might play a key role in this EC dysfunction [11, 12]. A recent study demonstrated the contribution of Wnt/β-catenin signaling to the regulation of the BBB permeability by affecting TJ proteins such as CLDN5 and Occludin (OCLN) in adult mice [13].

The β-catenin mediated Wnt signaling pathway leads to the recruitment of the β-catenin destruction complex upon binding of Wnt molecules to receptor protein Frizzled (Fzd) 4 and co-receptor low density lipoprotein receptor-related protein (Lrp) 5 or 6. This leads to the intracellular stabilization of β-catenin, resulting in its translocation to the nucleus. Once in the nucleus, β-catenin mediates the transcription of numerous genes involved in processes such as EC proliferation and differentiation, and TJ protein expression, implying an important role in the BBB [7, 14–16]. The Wnt/β-catenin signaling pathway is the most important pathway regulating the BBB in development, but seems to also play a role in adulthood [7, 17–20]. Thus, understanding the involvement of Wnt signaling in regulating TJ proteins in adult ECs might give insight into BBB pathology in diseases such as stroke and cSVD.

Of the 19 Wnt ligands, the regulation of BBB maturation is controlled by one of the most investigated Wnt ligands, namely Wnt7a. The interaction of Wnt7a with receptor Fzd4 and Lrp5/Lrp6 co-receptor controls brain angiogenesis and vessel formation by regulating endothelial tip cell formation [21]. In addition, Wnt7a mediated β-catenin activation regulates neural progenitor cell proliferation and differentiation [22]. Deletion of Wnt7a leads to major defects in CNS angiogenesis, while knocking out β-catenin resulted in similar angiogenic abnormalities [17]. Treatment with Wnt7a protein increases the expression of *Cldn5* in an immortalized brain endothelioma cell line (bEnd.3), leading to increased trans-endothelial electrical resistance (TEER) and decreased permeability of the endothelial monolayer following β-catenin activation [23–25]. The expression of CLDN5 was increased in both passage 1 and 3 ECs derived from human pluripotent stem cell (hPSC) in response to β-catenin activation, but the effects were less prominent in the later passage cells [25]. In passage 4 cells, β-catenin activation did not increase the number of ECs or CLDN5 levels, indicating a stage-dependent response to Wnt7a stimulation [25]. On the contrary, activation and nuclear accumulation of β-catenin resulted in the inhibition of *Cldn5* expression and promotion of angiogenesis [26, 27]. Thus, the molecular effects of Wnt7a mediated β-catenin activation on mature ECs and BBB integrity remains unclear.

The aim of this study was to investigate the underlying pathways determining in vitro effects of Wnt7a mediated β-catenin signaling on mature brain endothelial barrier integrity. The impact of Wnt7a stimulation on TJ protein expression mediated by β-catenin activation was investigated in bEnd.3 mouse brain ECs along with TEER measurements to investigate the effects on barrier function. Additionally, Gene ontology (GO) enrichment analysis of the transcriptomic signature was performed to identify modulators of this signaling pathway. GO enrichment analysis is used widely to interpret high throughout molecular data and to generate hypothesis about underlying biology. It represents a uniform vocabulary to specify cellular location, molecular function, and participation in biological process of human and model organisms [28]. Typically, analysis begins by identifying a list of differentially expressed genes. GO enrichment is then used to determine which GO terms are over- or under-represented with a gene set of interested in order to gain insights into the biological relevance of alterations in genes [29–34]. Results from these studies can then be used to support or refute hypotheses, inferences, or conclusions about the biology or evolution of the study system. We hypothesized that Wnt7a activates β-catenin-mediated Wnt signaling leading to regulation of TJ protein expression, affecting the barrier function of ECs. A clear understanding of this pathway might present a potential therapeutic target in repairing BBB damage in disease.

## Materials and Methods

### Cell Culture

The immortalized murine brain ECs, bEnd.3 cells (Cat no. CRL-2299, American Type Culture Collection, Manassas, VA, USA [35]) were cultured in Dulbecco’s Modified Eagle’s Medium high glucose (DMEM, Cat no. D6429, Merck Millipore, Burlington, MA, USA) supplemented with 10% fetal bovine serum (FBS) and 100 µg/mL penicillin/streptomycin (P/S) at 37 °C and in an atmosphere of 5% CO_2_. BEnd.3 cells between passage 26 and 28 were seeded onto multi-well plates, membrane inserts, or glass coverslips at a density of 1.5 × 10^4^ to 1.0 × 10^5^ cells/cm^2^ for expression and functional studies and treated at confluency (1–3 days after seeding).

Cells were treated with recombinant Wnt7a (Cat no. SRP3296, Sigma-Aldrich, St. Louis, MO, USA), dissolved in PBS containing 0.1% bovine serum albumin (BSA-PBS) to study the effects of paracrine mediated Wnt signaling on ECs. Briefly, cells were grown to confluency and treated with control (0.1% BSA-PBS) or Wnt7a (50 or 100 ng/ml in 0.1% BSA-PBS) for 24 h. These concentrations were used based on manufacturer’s ED_50_ and previous publications [36–39]. Wnt7a activation of β-catenin was investigated by co-administration with XAV939 (XAV, Cat no. X3004, Sigma-Aldrich), a Tankyrase inhibitor known to selectively inhibit Wnt/β-catenin mediated transcription [40, 41]. Briefly, cells were grown to confluency and treated with control, Wnt7a (100 ng/ml), XAV (10 µM) or a co-administrated of Wnt7a with XAV (100 ng/ml and 10 µM, respectively) for 24 h.

Hypoxia-inducible factor 1α (Hif1α) activation mediated by Wnt7a was investigated by silencing the *Hif1α* gene with siRNA. Briefly, cells were grown to confluency and transfected with 4 µg/ml Lipofectamine™ 2000 Transfection Reagent (Cat no. 11668, Invitrogen, Waltham, MA, USA) and 20 µM siRNA in DMEM high glucose containing 10% FBS and 10% Opti-MEM (Cat no. 31985070, ThermoFisher Scientific, Waltham, MA, USA) for 24 h. Negative control siRNA (Silencer™ Cy™3-labeled Negative Control No. 1 siRNA, Cat no. AM4621, Invitrogen), or a validated *Hif1α* siRNA (Silencer™ Select Pre-Designed mouse Hif1α siRNA, sequence 5’◊3’: Sense CCUUUACCUUCAUCGGGAAAtt; Antisense UUUCCGAUGAAGGUAAAGGag, Cat no. 4,390,771, Invitrogen) were used. After 24 h, cells were washed with PBS and treated with either control or Wnt7a (100 ng/ml) for 24 h. Cells were washed and fixed with 4% paraformaldehyde (PFA) for immunocytochemistry or lysed for RNA isolation, after respective treatment. Immunocytochemistry samples were kept at 4 °C and RNA samples at -20 °C until further use.

### Immunocytochemistry

Immunocytochemistry was performed after seeding bEnd.3 cells at a density of 1.5-5.0 × 10^4^ cells/cm^2^ on glass coverslips or on a 96-well plate. Cells were washed in PBS and fixed with 4% PFA for 10 min. at room temperature. Cells were then blocked in PBS blocking buffer containing 1% normal donkey serum and 0.3% Triton X-100 for 1 h. Then, cells were incubated with appropriate primary antibody (Supplementary Table 1) overnight at 4°C followed by incubation with appropriate secondary antibodies in blocking buffer (Supplementary Table 1) for 2 h. Lastly, a counterstaining of the nuclei with NucBlue (Cat no. R37605, Invitrogen) was performed and cells were mounted using antifading Mounting Medium (Prolong gold, Agilent Technologies, Santa Clara, CA, USA) before imaging.

### Image Acquisition and Analysis

Images were captured by an investigator masked to the treatment conditions using a confocal microscope (DMI 4000, Leica, Freiburg, Germany) or a fluorescent live cell imager (ImageXpress Pico Automated Cell Imaging System, Molecular Devices, San Jose, CA, USA). For confocal imaging, six image volumes (175 × 175 μm) were acquired with a 1 μm step size at a magnification of 63x. Images captured with the fluorescent live cell imager (690 × 690 μm) were acquired at a magnification of 40x. Subsequently, image stacks underwent maximal intensity projections and mean gray values and integrated density values of the resulting images were obtained using ImageJ software (National Institutes of Health, Bethesda, MD, USA). Three fields of view were acquired from every replicate to compare the different treatment conditions. In each field of view, the overall mean gray value and integrated density values were normalized to the number of cells. Additionally, for active β-catenin the average nuclear, cytoplasmic, and membrane concentrations were measured by averaging values of the mean intensity of three randomly selected cells per field of view. All quantifications were performed in ImageJ and expressed as intensity levels corrected versus control.

### Quantitative PCR (qPCR)

Total RNA was isolated using TRIzol Reagent (Invitrogen) according to the TRIzol method and stored at -80 °C before use. Quality and quantity were checked using NanoDrop 1000 spectrophotometer and the RNA was reverse transcribed into cDNA using the high-capacity RNA-to-cDNA kit (Cat no. 1,708,891, Bio-rad laboratories, Inc., Hercules, CA, USA) according to manufacturer’s manual. CDNA samples were stored at -20 °C before use. QPCR was performed using Sensimix™ SYBER® & Fluorescein kit (Cat no. QT615-05, Meridian Bioscience Inc., Cincinnati, OH, USA) on the Light Cycler 480 (Roche Applied Science, Penzberg, Germany) with the following qPCR program: 10 min. at 95 °C followed by 55 cycles a 10s at 95 °C and 20s at 60 °C. Temperature was increased from 60 to 95 °C for melting curve analyses. Primers were designed to cover exon-exon junctions and all possible splice variants using NCBI Primer-BLAST tool. Primers were synthesized by Eurofins Genomics (Ebersberg, Germany) and quality was ensured by testing on cell cultures, as well as by calculation of primer efficiency. At least two stable reference housekeeping genes were selected from a selection of three genes by using the GeNorm Software (Primerdesign, Southampton, NY, USA). Primers are listed in Supplementary Table 2. Gene expression analysis was performed using LinReg PCR (Ver. 2014.0) and the Light Cycler 480 data converter (Ver. 2014.1) and shown as fold change (FC) compared to control.

### RNA Sequencing

Total isolated RNA quantity was checked using Qubit 2.0 Fluorometer (Invitrogen) and RNA quality was assessed using Bioanalyzer (Cat no. RNA 6000 Nano kit; 2100 Bioanalyzer, Agilent Technologies). Purification of mRNA from total RNA (NEXTFLEX Poly(A) Beads 2.0, Cat no. NOVA-512,992, PerkinElmer, Waltham, MA, USA) and directional, strand-specific RNA library preparation (NEXTFLEX Rapid Directional RNA-Seq Kit 2.0, Cat no. NOVA-5198, PerkinElmer) was performed according to manufacturer’s protocol. Sequencing was performed using NovaSeq 6000 Sequencing system (NovaSeq S Prime flow cell 200 cycles; NovaSeq 6000, Illumina, Inc, San Diego, CA, USA) according to manufacturer’s protocol. The raw sequencing data was trimmed using *fastp*, remaining reads were then mapped against the Ensembl mouse genome (release 100) using STAR (version 2.7.3a) and quantified using RSEM (v.1.3.1). The resulting raw read counts were processed using the R package DESeq2. Genes with were not sequenced (0 reads) in more than 75% of the samples of any given condition were removed. Genes were considered differentially expressed with an adjusted *p*-value (false discovery rate; FDR) below 0.01. Gene ontology (GO) enrichment analysis was performed using g:Profiler [42] and the Database for Annotation, Visualization, and Integrated Discovery (DAVID) v6.8 [43, 44]. The modified Fisher exact *p*-value (EASE score) < 0.05 and FDR < 0.05 were considered enriched. Gene interaction network analysis was performed using Cytoscape version 3.9.1 [45].

### Trans Endothelial Electrical Resistance (TEER) Assay

TEER was monitored every 2–3 days using an epithelial volt ohm meter (EVOM-2, WPI, UK) connected to an electrode (STX4 EVOM, Cat no. EVM-EL-03-03-01, WPI). Experiments were performed using PET membrane inserts (Cat no. 353,095, Falcon) with an insert diameter of 6.4 mm and pore diameter of 0.4 μm in combination with a 24-well companion plate (Cat no. 353,504, Falcon). Inserts were coated with rat tail collagen I (100 µg/ml, Cat no. 3440-005-01, R&D Systems, Minneapolis, MN, USA) for 1 h at 37 °C. Before seeding cells, resistance measurements were performed on all coated wells to determine the blank resistance. Measurements were performed on a heating surface to maintain a stable temperature of 37 °C during measurements. BEnd.3 cells were plated on the membrane inserts at a density of 4.5 × 10^4^ cells/cm^2^ and left to attach and reach full confluency for 3 days. Treatment with either control or 100 ng/ml Wnt7a was initiated at day 3 and refreshed every 2–3 days before measurements. The TEER was calculated using the following formula:

$$TEER\,\,(\Omega \,*\,c{m^2})\, = \,({R_{total}}\, - \,{R_{blank}})\,*Insert\,membrane\,area$$

### Statistical Analysis

Data was analysed using GraphPad Prism 9 (Dotmatics). Data distribution was tested using Shapiro-Wilk test for normality. Unpaired Student t-tests were used to compare Wnt7a vs. control. One-way ANOVA or Kruskal-Wallis test (for non-parametric data) with post-hoc Tukey’s multiple comparisons test was used to assess multiple comparisons. *P* < 0.05 was considered statistically significant and data are expressed as mean ± SEM.

## Results

### Wnt7a Leads to Increased Levels of Nuclear Active β-Catenin

**Fig. 1:**
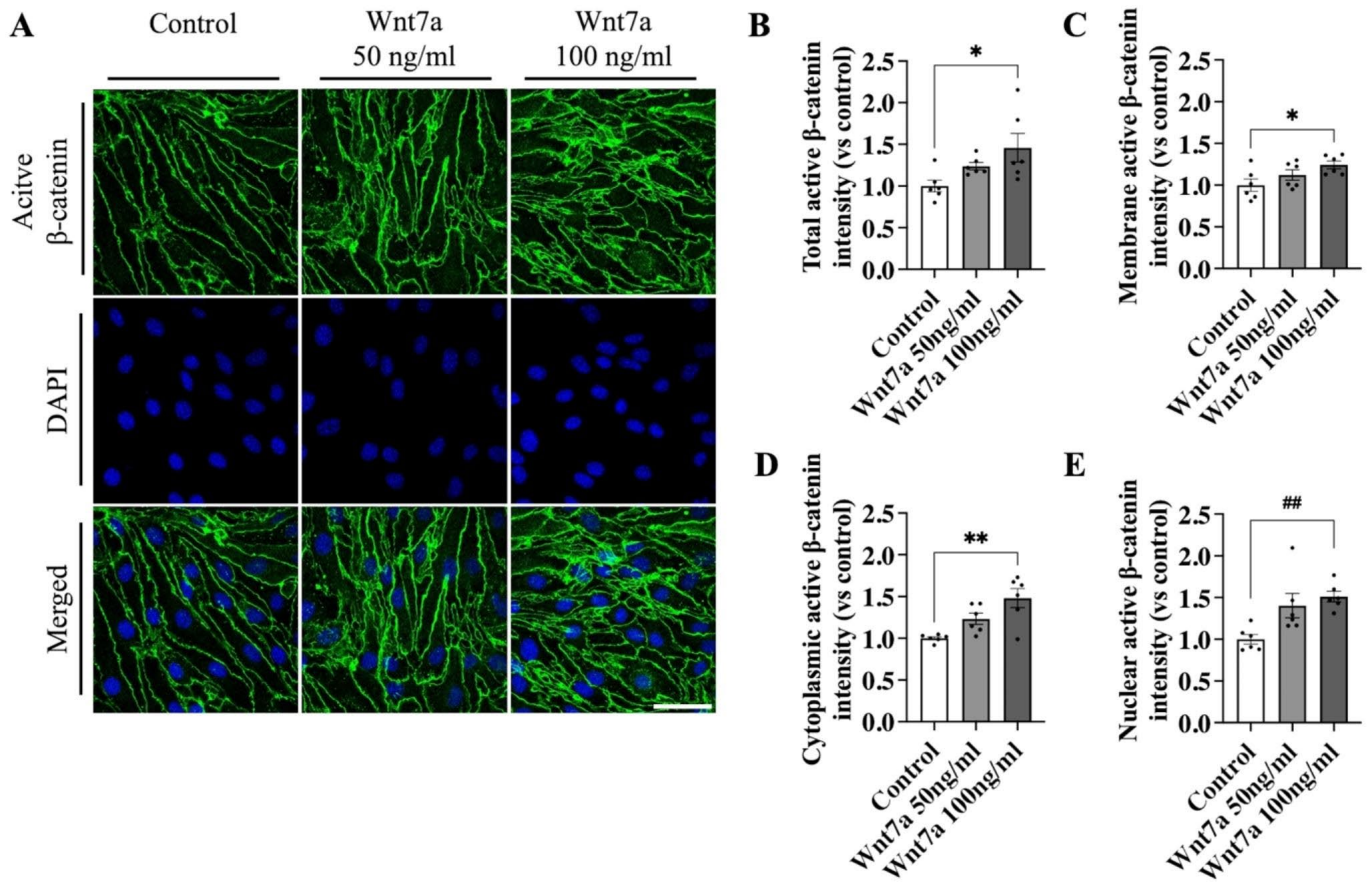
Wnt7a led to increase in active β-catenin levels in bEnd.3 cells in vitro. **A** Immunocytochemistry of active β-catenin after treatment with either 50 or 100 ng/ml recombinant Wnt7a. Images were acquired at a magnification of 63x; Scale bar, 50 μm. **B** Quantification of relative active β-catenin intensity revealed an increase in total active β-catenin in bEnd.3 cells when treated with 100 ng/ml Wnt7a. **C** Membrane bound active β-catenin was increased in cells treated with 100 ng/ml Wnt7a and in the **D** cytoplasm and **E** nucleus. Treatment with 50 ng/ml did not lead to significant changes in active β-catenin. Graph represents mean ± SEM; n = 6; *p < 0.05, **p < 0.01, One-way ANOVA with post-hoc Tukey’s multiple comparisons; **##***p* < 0.01, Kruskal-Wallis with post-hoc Dunn’s multiple comparisons

To investigate whether Wnt7a stimulation causes β-catenin mediated signaling in a mouse brain EC line, we performed immunocytochemistry on bEnd.3 cells treated with recombinant Wnt7a protein for 24 h (Fig. 1A). The overall signal intensity of the active form of β-catenin was quantified in addition to the signal intensity in the membrane, cytoplasm, and nucleus. A significant increase in overall active β-catenin intensity was observed when cells were treated with 100 ng/ml Wnt7a compared to control (1.46 ± 0.17 FC, *p* = 0.025). Similar increases in active β-catenin were observed in the membrane (1.24 ± 0.05 FC, *p* = 0.036), cytoplasm (1.48 ± 0.11 FC, *p* = 0.001), and nucleus (1.52 ± 0.06 FC, *p* = 0.002). Treatment with 50 ng/ml Wnt7a, however, did not lead to a significant increase in overall active β-catenin intensity (1.24 ± 0.04 FC, *p* = 0.305), as well as in membrane (1.12 ± 0.06 FC, *p* = 0.370), cytoplasmic (1.23 ± 0.07, *p* = 0.116), or nuclear intensity (1.40 ± 0.15 increase, *p* = 0.092) compared to control. These results indicate that 100 ng/ml Wnt7a was effective to promote β-catenin activation.

Next, we investigated whether XAV, a known Tankyrase inhibitor, was able to inhibit the Wnt7a-mediated increase in active β-catenin. XAV inhibits the PARylation of Axin, which is one of the proteins in the β-catenin destruction complex. Tankyrase mediated PARylation of Axin leads to the ubiquitination and degradation of this protein. Thus, by inhibiting Trankyrase, the β-catenin destruction complex can assemble and lead to the ubiquitination and degradation of β-catenin [46].

**Fig. 2:**
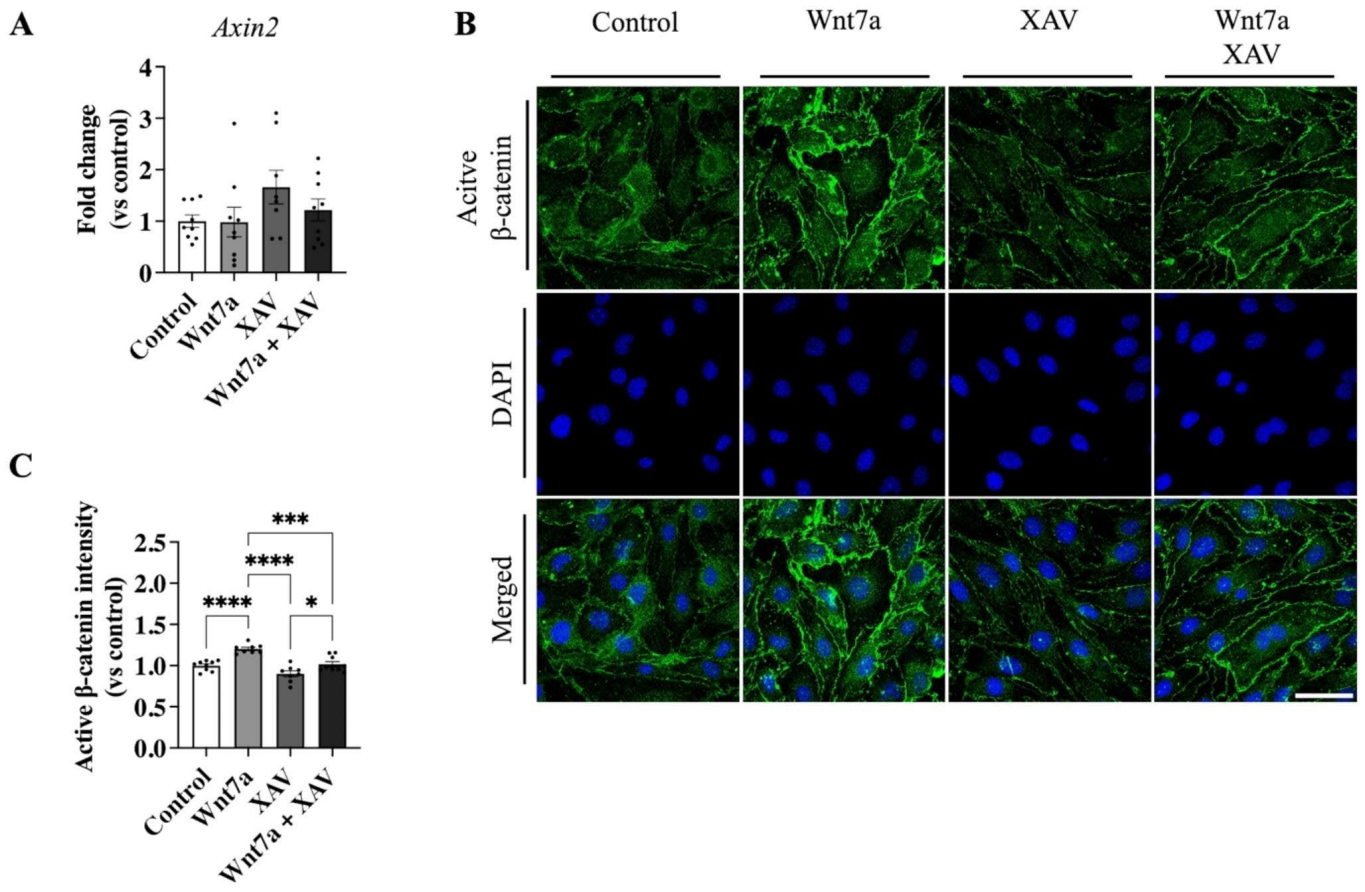
XAV inhibited the activation of β-catenin in response to Wnt7a treatment. **A** In vitro Wnt7a treatment did not lead to any changes in the β-catenin target gene *Axin2* mRNA expression **B** Immunocytochemistry of active β-catenin in bEnd.3 cells treated with either control, 100 ng/ml Wnt7a, 10 µM XAV, or co-treatment with Wnt7a and XAV. Images were acquired at a magnification of 63x; Scale bar, 50 μm. **C** The overall total β-catenin activation mediated by Wnt7a was significantly inhibited by XAV. XAV led to decreased active β-catenin levels compared to control. Graph represents mean ± SEM; n = 8–9; *p < 0.05, ***p < 0.001, ****p < 0.0001; One-way ANOVA with post-hoc Tukey’s multiple comparisons

Treatment with 100 ng/ml Wnt7a did not lead to a significant increase in the transcription of *Axin2* (0.98 ± 0.29 FC, *p* > 0.99; Fig. 2A), a direct target of the Tcf/LEF factor mediated Wnt pathway [47]. Inhibition of the active β-catenin with XAV also did not lead to any significant changes in *Axin2* mRNA expression (1.66 ± 0.33 FC, *p* = 0.26; Fig. 2A). However, overall β-catenin activity, assessed by immunocytochemistry (Fig. 2B), showed a significant increase in active β-catenin in cells treated with 100 ng/ml Wnt7a compared to controls (1.20 ± 0.02 FC, *p* < 0.0001; Fig. 2C). The increase in active β-catenin was not observed when cells were treated with XAV (0.90 ± 0.03 FC compared to control, *p* = 0.07; Fig. 2C) or when cells were co-treated with Wnt7a and XAV (1.02 ± 0.03 FC compared to control, *p* = 0.96; Fig. 2C). Total active β-catenin levels in Wnt7a treated cells were significantly increased compared to co-treatment with Wnt7a and XAV (*p* = 0.0002; Fig. 2C), indicating the ability of XAV to inhibit the Wnt7a mediated activation of β-catenin.

### Wnt7a Stimulation Leads to Reduced Endothelial Barrier Function by Decreasing Tight Junction Protein Claudin-5 Via β-Catenin Mediated Signaling

**Fig. 3:**
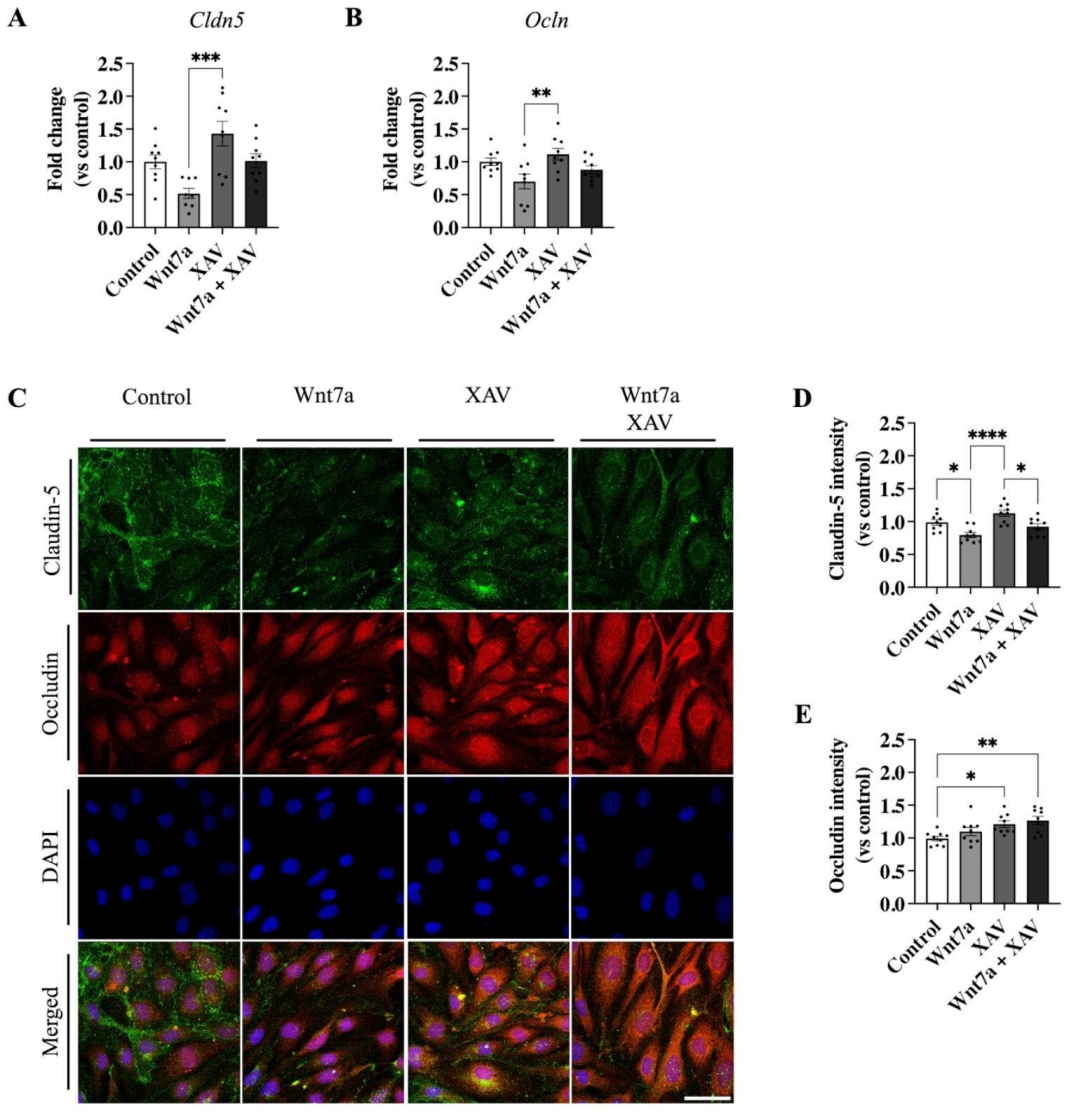
Modulation of β-catenin led to changes in TJ proteins Claudin-5 and Occludin. **A** In vitro activation of β-catenin mediated by Wnt7a led to downregulation of *claudin-5* and **B***occludin* mRNA. **C** Immunocytochemistry of Claudin-5 or Occludin in bEnd.3 cells treated with either control, 100 ng/ml Wnt7a, XAV, or co-treatment with Wnt7a and XAV. Images were acquired at a magnification of 63x; Scale bar, 50 μm. **D** Decreased levels of Claudin-5 protein were mediated by wnt7a activation of β-catenin. **E** inhibition β-catenin levels by XAV led to a significant increase in Occludin compared to control. However, Wnt7a did not decrease levels of Occludin. Abbreviations: Cldn5 = claudin-5; Ocln = Occludin. Graph represents mean ± SEM; n = 8–9, *p < 0.05, **p < 0.01, ***p < 0.001, ****p < 0.0001; One-way ANOVA with post-hoc Tukey’s multiple comparisons

The effects of Wnt7a signaling on the TJ proteins *Cldn5* and *Ocln* mRNA expression and protein levels mediated by β-catenin activation were investigated with qPCR and immunocytochemistry, respectively (Fig. 3). Wnt7a stimulation led to a significant decrease in both *Cldn5* (*p* = 0.0002; Fig. 3A) and *Ocln* (*p* = 0.007; Fig. 3B) mRNA expression levels compared to cells in which β-catenin was inhibited with XAV. Similar results were observed for protein levels of CLDN5, which was significantly decreased by Wnt7a stimulation compared to control (0.80 ± 0.04 FC, *p* = 0.022; Fig. 3D). No difference in protein levels of CLDN5 was observed when Wnt7a and XAV were co-administered compared to control (0.92 ± 0.05 FC, *p* = 0.730; Fig. 3D). Despite reducing *Ocln* mRNA levels, Wnt7a treatment did not lead to changes in OCLN protein levels compared to control (1.10 ± 0.06 FC, *p* = 0.483; Fig. 3E). However, treatment with XAV, and co-treatment with Wnt7a and XAV, significantly increased protein levels of OCLN compared to control (1.21 ± 0.05 FC and 1.27 ± 0.06 FC, *p* = 0.031 and *p* = 0.005, respectively; Fig. 3E). These results indicate that β-catenin mediated the effects of Wnt7a stimulation on TJ proteins, which are responsible for EC barrier function.

**Fig. 4:**
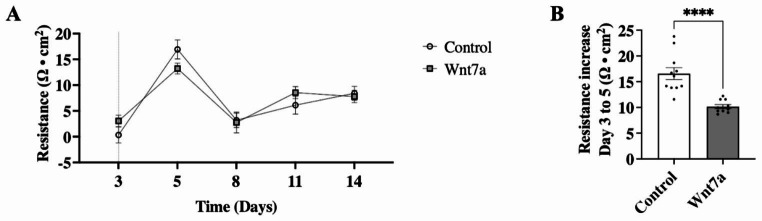
Wnt7a led to a reduction in formation of the endothelial barrier. **A** Trans endothelial electrical resistance (TEER) measurements of the bEnd.3 monolayer in vitro. No differences in electrical resistance were observed after 14 days of Wnt7a treatment compared to control. Dotted vertical line indicates start of Wnt7a treatment. **B** Wnt7a blunted the increase in the electrical resistance between day 3 to day 5 compared to control. Graph represents mean ± SEM; n = 10–11, ****p < 0.0001; Unpaired students t-test

The EC barrier integrity was studied by measuring the electrical resistance of the endothelial monolayer in culture by TEER. The establishment of the BBB is characterized by the endothelial barrier formation comprised of TJ proteins tightly connecting the ECs, resulting in an increased electrical resistance over time [48]. No overall differences were observed in electrical resistance between cultures treated with Wnt7a protein and control over a period of 14 days (Fig. 4A). However, a significant increase in resistance from day 3 to day 5 was observed, with Wnt7a treatment leading to a reduced increase in resistance compared to control (10.17 ± 0.39 vs. 16.58 ± 1.15 Ω, *p* < 0.0001; Fig. 4B). These results suggest that Wnt7a stimulation inhibits endothelial barrier formation.

### *Hif1α* Activation in Response to β-Catenin Mediated Wnt7a Signaling is Not Involved in the Decrease of Tight Junction Proteins Claudin-5 and Occludin

**Fig. 5:**
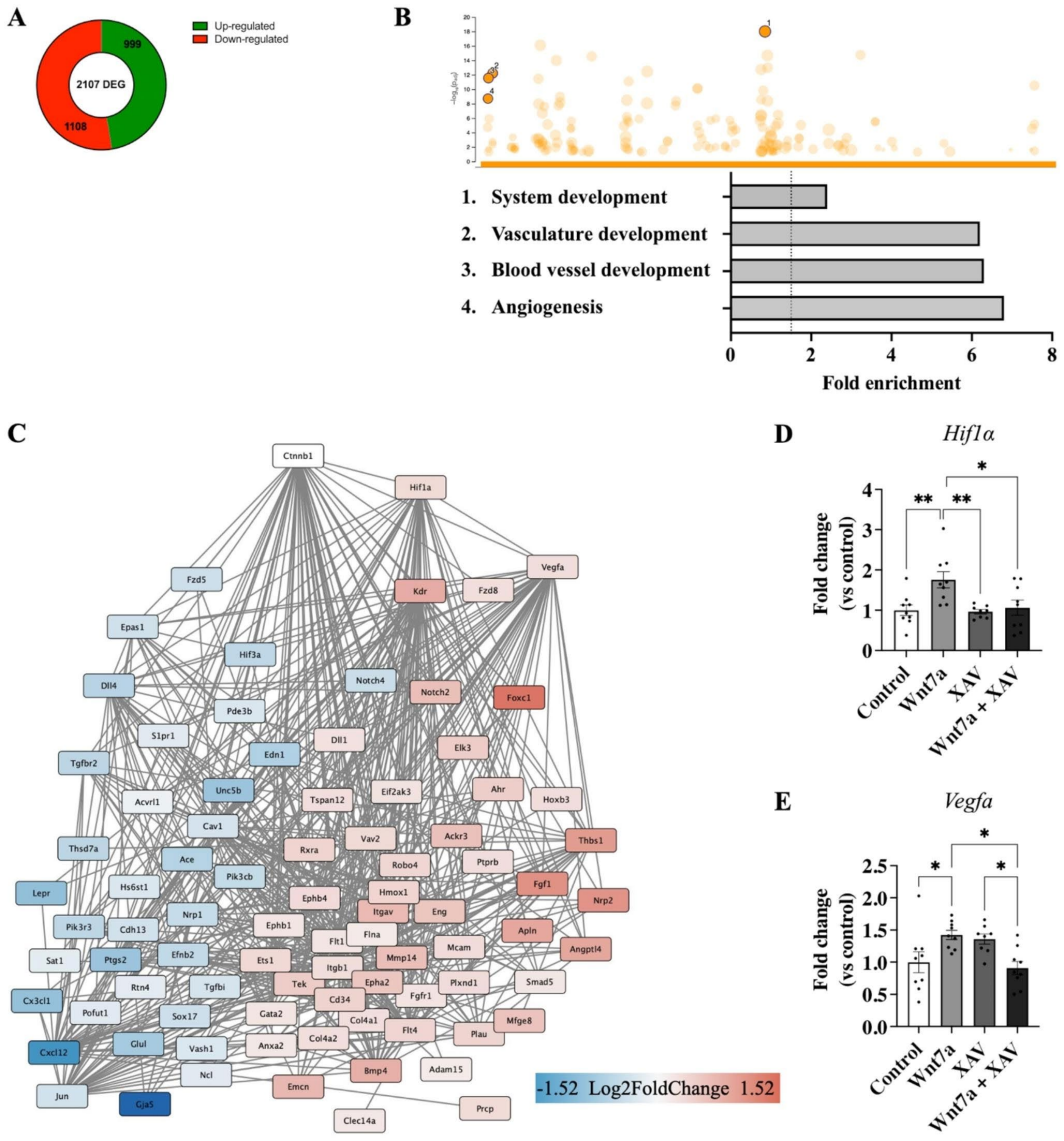
Wnt7a led to regulation of 2,017 differentially expressed genes involved in e.g. angiogenesis. **A** Wnt7a treatment led to changes in 2,017 differentially expressed genes (DEG) in bEnd.3 cells (n = 3) **B** Gene Ontology (GO) enrichment analysis revealed the enrichment of biological processes such as vascular development, blood vessel development, and angiogenesis. **C** Analysis of Wnt7a regulated genes mediated by β-catenin in the angiogenesis pathway revealed the upregulation of *Hif1α and Vegfa*. **D** The upregulation of *Hif1α* and **E***Vegfa* by Wnt7a was mediated by β-catenin (n = 8–9). Graph represents mean ± SEM; *p < 0.05, **p < 0.01; One-way ANOVA with post-hoc Tukey’s multiple comparisons

To decipher the molecular mechanisms involved in the response of brain ECs to Wnt7a stimulation, RNA sequencing was performed to assess their transcriptomic regulation. This revealed 2,107 differently expressed genes (DEG) when comparing Wnt7a to control treatment (Fig. 5A). GO enrichment analysis of the top 100 DEG revealed enriched pathways such as vasculature development (6.2-fold enriched), blood vessel development (6.3-fold enriched) and angiogenesis (6.8-fold enriched, Fig. 5B). Pathway analysis of the genes mediated by β-catenin in the angiogenesis pathway showed upregulation of *Hif1α* and vascular endothelial growth factor A (*Vegfa)* (0.28 and 0.26 log2FC, respectively), genes typically involved in response to hypoxia (Fig. 5C) [49]. Quantification of mRNA levels in cells treated with Wnt7a showed an increase in both *Hif1α* and *Vegfa* compared to control (1.76 ± 0.20 and 1.42 ± 0.07 FC, *p* = 0.010 and *p* = 0.047; Fig. 5D and E respectively).

**Fig. 6:**
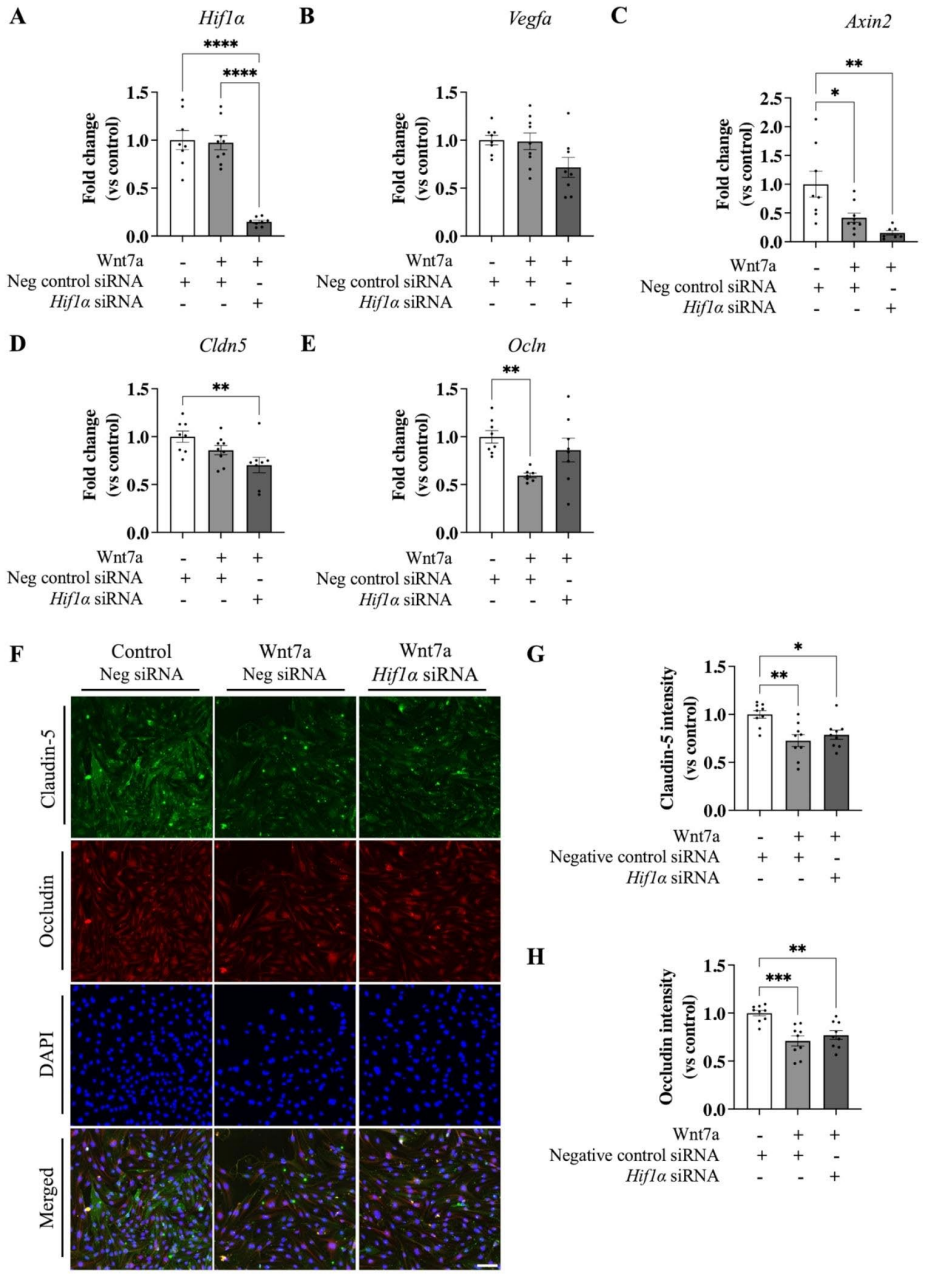
Wnt7a regulation of tight junction proteins Claudin-5 and Occludin was not mediated by *Hif1α*. **A** Transfection with *Hif1α* siRNA led to the downregulation of *Hif1α*. **B** Wnt7a did not change *Vegfa* mRNA expression, with no effect of *Hif1α.***C** Wnt7a led to the significant downregulation of the β-catenin target gene *Axin2*, which was not mediated by *Hif1α*. **D***Claudin-5* was downregulated by Wnt7a only when *Hif1α* was silenced, **E** while *Occludin* was normalized by silencing *Hif1α* in the present of Wnt7a. **F** Immunocytochemistry of Claudin-5 or Occludin in bEnd.3 cells treated with either control and negative control siRNA, 100 ng/ml Wnt7a and negative control siRNA, or 100 ng/ml Wnt7a and *Hif1α* siRNA. Images were acquired at a magnification of 40x; Scale bar, 100 μm. **G** Decreased Claudin-5 protein level by Wnt7a was not mediated by *Hif1α*. **H** Similar results were observed for Occludin. Abbreviations: Neg = negative; Cldn5 = claudin-5; Ocln = Occludin. Graph represents mean ± SEM; n = 7–9, *p < 0.05, **p < 0.01, ***p < 0.001, ****p < 0.0001; One-way ANOVA with post-hoc Tukey’s multiple comparisons

The contribution of *Hif1α* activation in regulating TJ proteins was investigated by silencing the *Hif1α* gene with a validated commercially available siRNA. *Hif1α* was significantly downregulated when treated with Wnt7a and *Hif1α* siRNA compared to Wnt7a and negative control siRNA (0.14 ± 0.02 FC, *p* < 0.0001; Fig. 6A). No differences were found in *Vegfa* expression when treated with either Wnt7a or co-treated with Wnt7a and *Hif1α* siRNA (Fig. 6B). The expression of *Axin2* was significantly downregulated by Wnt7a (0.42 ± 0.08 FC, *p* = 0.019; Fig. 6C), with no difference compared to when treated with Wnt7a and *Hif1α* siRNA (*p* = 0.42). *Cldn5* expression was significantly downregulated by Wnt7a only when inhibiting *Hif1α* mRNA (0.70 ± 0.08 FC, *p* = 0.01; Fig. 6D), while *Ocln* expression was downregulated by Wnt7a treatment alone (0.59 ± 0.02 FC, *p* = 0.009; Fig. 6E).

When *Hif1α* was silenced in cells treated with Wnt7a, *Ocln* mRNA levels normalized compared to control (0.86 ± 0.12 FC, *p* = 0.48; Fig. 6E), suggesting that HIF1α signaling might play a role in regulating the effects of Wnt7a on endothelial barrier function. Protein levels of CLDN5 and OCLN were investigated after *Hif1α* knockdown by immunocytochemistry (Fig. 6F). Consistent with our previous data (Fig. 3D), treatment with Wnt7a reduced levels of CLDN5 protein compared to control (0.73 ± 0.06 FC, *p* = 0.002; Fig. 6G). However, no differences in CLDN5 levels were observed between Wnt7a treated cells treated with either negative control or *Hif1α* siRNA (*p* = 0.68). Similar effects on OCLN levels were observed, with Wnt7a treatment inducing a reduction compared to control (0.71 ± 0.05 FC, *p* = 0.0003; Fig. 6H), and with no differences observed between the negative control and *Hif1α* siRNA treated cells treated with Wnt7a (*p* = 0.59). These results show that HIF1α signaling does not directly interact with Wnt7a signaling in regulating the expression of TJ proteins CLDN5 and OCLN but might modulate gene expression (Fig. 6D-E).

## Discussion

The aim of this study was to investigate the effects of the Wnt7a/β-catenin signaling pathway on EC barrier function. We showed that 100 ng/ml Wnt7a increased protein levels of active β-catenin in the cytoplasm, membrane, and nucleus of the bEnd.3 EC line. This increase was reversed when degradation of the destruction complex was prevented by the Tankyrase inhibitor XAV, validating Wnt7a mediated activation of β-catenin signaling. Of note, increased β-catenin activation did not translate to an increase in expression of its target gene *Axin2*, implying that other factors might influence the transcription of the downstream β-catenin target genes, in response to Wnt7a stimulation. This Wnt7a/β-catenin signaling had functional significance since Wnt7a stimulation impaired EC barrier formation via reduced expression of the TJ protein, CLDN5. Endothelial genes commonly activated in response to hypoxia, *Hif1α* and *Vegfa*, were upregulated by Wnt7a activation of β-catenin. Interestingly, other studies have described the expression of Wnt7a following hypoxic conditions, indicating that the interplay of these signaling molecules might contribute to hypoxia-induced events, during pathological conditions such as stroke or cSVD [50]. However, decreases in TJ proteins in CLDN5 and OCLN were not mediated by *Hif1α* in our study. Overall, our analysis of TJ protein expression and EC barrier function suggest the involvement of Wnt7a in increasing the permeability of ECs, an effect that could have consequences for the endothelial barrier function.

Activation of β-catenin signaling by Wnt7a has been shown to depend on the cell type and developmental stage within the CNS. Activation of Wnt7a in the developing hippocampus triggers β-catenin mediated signaling, leading to positive influences on synaptogenesis [51].

Here, β-catenin mediated signaling by Wnt7a is enhanced by the presence of receptor co-factors Gpr124 and Reck [21]. However, β-catenin is not involved in all aspects of Wnt7a function at the synapse. For example, localization of Wnt7a in mouse cerebellar synapses increased the size and spreading of axonal growth cones, and was essential for neurotransmitter release via actions on the cytoskeleton that do not involve β-catenin [52]. These findings suggest that Wnt7a might activate both β-catenin dependent and independent signaling, or one or the other, depending on environmental factors. Based on our findings that Wnt7a increases levels of active β-catenin, the ability of XAV to reverse Wnt7a effects, and evidence from previous work, we suggest that the effects of Wnt7a on brain ECs are predominantly mediated via β-catenin signaling. However, β-catenin independent signaling effects might also play a role [21, 51, 53]. In addition to confirming the role of the Wnt7a/β-catenin pathway, our findings highlight XAV as a potent molecule to modulate Wnt7a induced β-catenin signaling in brain ECs.

In our study, Wnt7a activation of β-catenin in vitro led to a decrease in the TJ proteins CLDN5 and OCLN in mouse brain ECs (bEnd.3 cells). However, previous studies have shown β-catenin mediated increases in TJ proteins. Stabilization of β-catenin via glycogen synthase kinase 3β (GSK-3β) inhibitor CHIR99021 in hPSC, or LiCl in an immortalized human brain microvascular EC line (hCMEC/D3), led to the upregulation of CLDN5 [13, 23]. Similarly, in vivo deletion of β-catenin in the brain endothelium led to decreased protein levels of both CLDN5 and OCLN in the cerebral cortex of mice [25]. Wnt7a derived from oligodendrocyte precursor cell (OPC) conditioned medium showed increased β-catenin and *Cldn5* expression in bEnd.3 cells, while siRNA knock down of Wnt7a in OPCs blocked these effects [54]. Conversely, stabilization of β-catenin with LiCl in ECs derived from murine embryonic stem cells showed a significant reduction in *Cldn5* mRNA and protein levels [27]. Here, translocation of β-catenin to the nucleus led to its binding to Foxo1, forming a Foxo1–β-catenin–Tcf complex at the *Cldn5* gene promotor site that inhibited its expression [27]. A correlation was observed between increased β-catenin and decreased *CLDN5* expression in both patient glioma tissue and malignant glioma cells lines [55]. The expression of β-catenin gradually increased in higher glioma tumor grades, while the expression of TJ proteins CLDN1 and CLDN5 were both decreased [55].

There is some discrepancy in the literature regarding the Wnt7a/β-catenin regulation of TJ proteins. For example, an in vitro study in bEnd.3 cells detected significant increases in levels of BBB-specific influx transporters, but did not observe changes in the expression of TJ proteins such as Ocln [17]. Functional effects of Wnt7a/β-catenin have also been examined. For example, an in vitro bEnd.3 permeability assay using Evans blue dye, decreased endothelial permeability in cells treated with Wnt7a compared to control treated cells [54]. Similarly, CHIR99021 treated hPSC showed increased TEER resistance and decreased permeability to a small molecule tracer, sodium fluorescein [25]. However, primary mouse brain ECs treated with Wnt3a, another activator of the β-catenin dependent Wnt pathway, did not produce an effect on EC permeability [56]. These findings contrast with the decrease in TEER values we observed after a 2-day stimulation with Wnt7a. Of note, results similar to ours were observed in a study of human aortic ECs where similar β-catenin mediated increases in permeability were detected [57].

The observed decrease in TEER values in both Wnt7a and control treated cells after 5 days may be explained by a model of EC function in which β-catenin levels must be maintained above a certain threshold level of β-catenin to ensure barrier function in adult CNS vasculature [58]. In this context, the β-catenin signaling response of bEnd.3 cells treated with 100 ng/ml Wnt7a might decrease after repeated stimulations due to desensitization, leading to disabled tight barrier maintenance and a decrease in electrical resistance. In support of this idea, previous studies suggest that activation of β-catenin due to phosphorylation and internalization of LRP6, leads to desensitization [59, 60].

Modulation of EC barrier permeability might be a crucial step towards EC proliferation and the initiation of brain angiogenesis through EC sprouting, both of which are processes known to require Wnt signaling [17, 21, 61]. An interesting factor that might modulate the angiogenic response of ECs is hypoxia induced transcription factor 1 α (HIF1α). Our data suggest that β-catenin activation through Wnt7a can increase the expression of *Hif1α* in ECs. HIF1α can regulate Wnt signaling and be the target of Wnt induced regulation [62–64], whilst hypoxia and HIF1α signaling regulates Wnt/β-catenin signaling in a cell- and developmental stage-specific manner [65, 66]. It is also suggested that these signaling pathways might have an indirect interaction rather than exerting direct regulation [64]. On the other hand, nuclear β-catenin/Tcf complex formation has been shown to induce *Hif1α* expression [63]. These results indicate that Wnt/β-catenin signaling can affect the expression of *Hif1α* to modulate the cellular response to events such as hypoxia. Our data support this notion of Wnt7a/β-catenin mediating the expression of *Hif1α*. However, in our study, the silencing of *Hif1α* with siRNA did not reverse the Wnt7a/β-catenin mediated downregulation of TJ proteins CLDN5 and OCLN. Interestingly, mRNA levels of *Ocln* were normalized by inhibiting *Hif1α*, while OCLN proteins levels were increased independently from β-catenin, suggesting that these signaling pathways might interact and indirectly regulate the endothelial barrier function. Thus, Wnt/HIF1α signaling in mature ECs may not be critical for barrier formation, but may play an indirect role in a context-dependent manner, for example via HIF1α/VEGFA signaling during hypoxic conditions in diseases such as stroke and cSVD. There was some discrepancy regarding the regulation of OCLN by Wnt7a in two of our data sets (Fig. 3E vs. 6H). On one hand (Fig. 3E), OCLN was not changed by Wnt7a stimulation while blocking β-catenin with XAV had a positive effect. On the other hand (Fig. 6H), OCLN was downregulated by Wnt7a stimulation and unchanged by *Hif1α* silencing. While these data are contradicting, it also indicates that the Wnt7a/β-catenin signaling can lead to HIF1α independent regulation of OCLN.

**Fig. 7:**
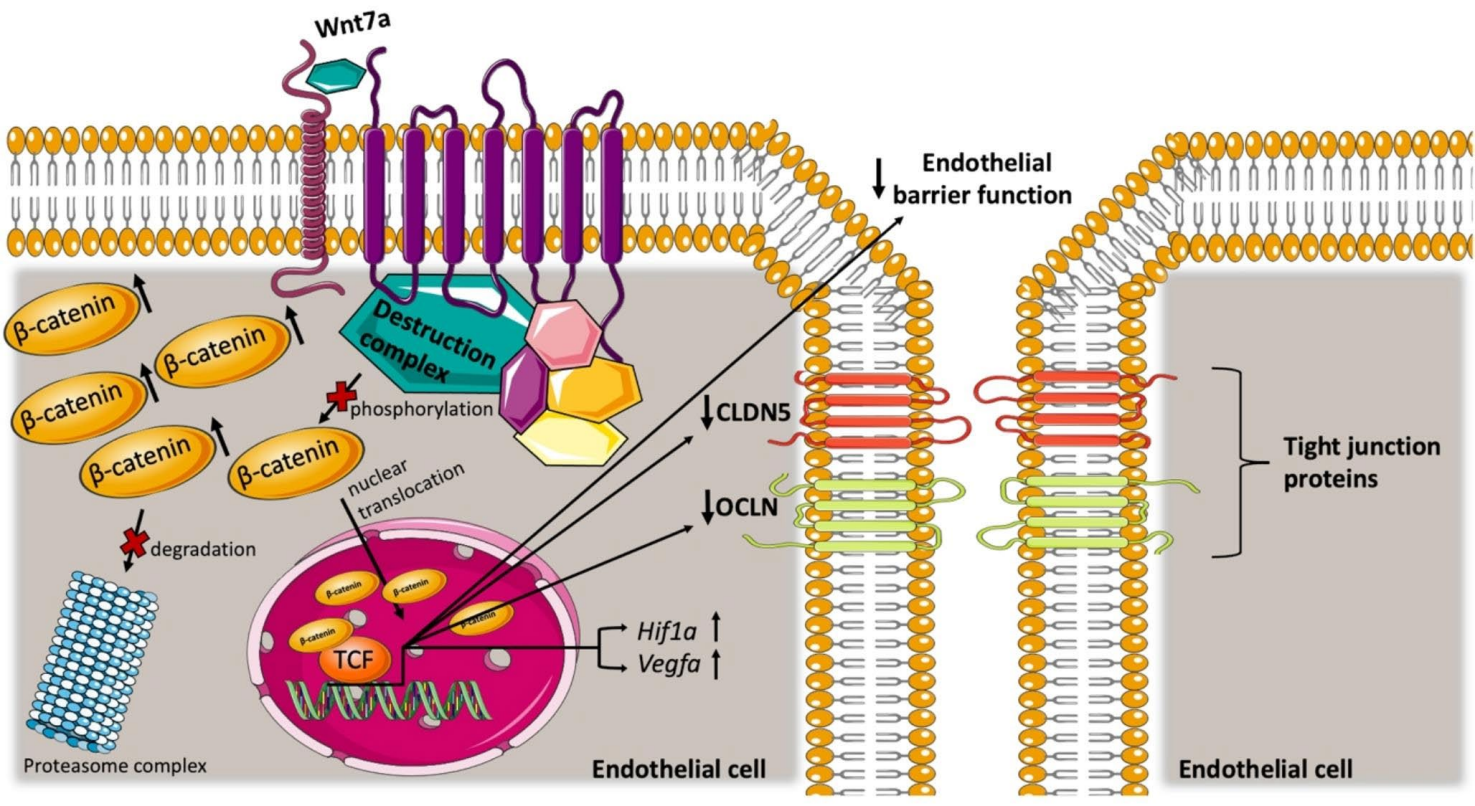
Schematic overview of our study findings proposed Wnt7a signaling pathway mechanism in bEnd.3 endothelial cells. Wnt7a binding to its receptors leads to activation of β-catenin by inhibiting its degeneration. This in turn results in the accumulation and nuclear translocation of β-catenin. Activation of this signaling pathway causes a decrease in the expression of tight junction proteins such as Claudin-5 (CLDN5) and a decreased endothelial barrier function. Activation of this signaling pathway also leads to an increase in *Hif1a* and *Vegfa* mRNA expression, which does not interact with the Wnt7a mediated regulation of the tight junction proteins. Abbreviations: CLDN5 = Claudin-5; OCLN = Occludin; Hif1a = hypoxia induced transcription factor 1 α; Vegfa = vascular endothelial growth factor A

In conclusion, we suggest that Wnt7a activates the β-catenin mediated Wnt signaling pathway, causing nuclear translocation of β-catenin, suppression of TJ protein expression, and ultimately a decrease in EC barrier function (illustrated in Fig. 7). Furthermore, we propose that these changes in EC properties are associated with events such as endothelial proliferation and angiogenesis, which are stimulated by Wnt7a signaling. Wnt7a/β-catenin mediated regulation of TJ proteins occurs independently of the Wnt/HIF1α signaling pathway. This pathway may play other roles in inducing angiogenesis in response to environmental factors such as hypoxia. However, future studies are needed to determine the specific role of HIF1α in modulating the Wnt/β-catenin signaling pathway. Understanding the role of Wnt/β-catenin signaling in hypoxia might lead to a better understanding of the cellular mechanisms involved in diseases such as cSVD.

### Electronic Supplementary Material

Below is the link to the electronic supplementary material.

Supplementary Material 1

## Data Availability

The authors confirm that the data supporting the findings of this study are available within the article and its supplementary material. Raw data are available from the corresponding author, upon request. Supplementary information is available at *Molecular Neurobiology* online.

## References

[1] Haddad-Tóvolli R, Dragano NRV, Ramalho AFS, Velloso LA (2017). Development and function of the blood-brain barrier in the Context of Metabolic Control. Front Neurosci.

[2] Kadry H, Noorani B, Cucullo L (2020). A blood–brain barrier overview on structure, function, impairment, and biomarkers of integrity. Fluids Barriers CNS.

[3] Quick S, Moss J, Rajani RM, Williams A (2021). A vessel for change: endothelial dysfunction in Cerebral Small Vessel Disease. Trends Neurosci.

[4] Liu W-Y, Wang Z-B, Zhang L-C (2012). Tight Junction in blood-brain barrier: an overview of structure, regulation, and Regulator substances. CNS Neurosci Ther.

[5] Bhat AA, Uppada S, Achkar IW (2019). Tight Junction proteins and Signaling pathways in Cancer and inflammation: a functional crosstalk. Front Physiol.

[6] Anderson JM, Itallie CMV (2009). Physiology and function of the tight Junction. Cold Spring Harb Perspect Biol.

[7] Liebner S, Corada M, Bangsow T (2008). Wnt/β-catenin signaling controls development of the blood–brain barrier. J Cell Biol.

[8] Greene C, Hanley N, Campbell M (2019). Claudin-5: gatekeeper of neurological function. Fluids Barriers CNS.

[9] Rouhl RPW, Damoiseaux JGMC, Lodder J (2012). Vascular inflammation in cerebral small vessel Disease. Neurobiol Aging.

[10] Hawkins BT, Davis TP (2005). The Blood-Brain Barrier/Neurovascular Unit in Health and Disease. Pharmacol Rev.

[11] Manukjan N, Ahmed Z, Fulton D (2020). A systematic review of WNT signaling in endothelial cell oligodendrocyte interactions: potential relevance to Cerebral Small Vessel Disease. Cells.

[12] Galatius S, Wroblewski H, Sørensen VB (1999). Endothelin and Von Willebrand factor as parameters of endothelial function in idiopathic dilated cardiomyopathy: different stimuli for release before and after heart transplantation?. Am Heart J.

[13] Hussain B, Fang C, Huang X (2022). Endothelial β-Catenin Deficiency causes blood-brain barrier breakdown via enhancing the Paracellular and transcellular permeability. Front Mol Neurosci.

[14] Foulquier S, Daskalopoulos EP, Lluri G (2018). WNT signaling in Cardiac and Vascular Disease. Pharmacol Rev.

[15] Clevers H (2006). Wnt/β-Catenin signaling in Development and Disease. Cell.

[16] Cattelino A, Liebner S, Gallini R (2003). The conditional inactivation of the β-catenin gene in endothelial cells causes a defective vascular pattern and increased vascular fragility. J Cell Biol.

[17] Daneman R, Agalliu D, Zhou L (2009). Wnt/β-catenin signaling is required for CNS, but not non-CNS, angiogenesis. Proc Natl Acad Sci.

[18] Stenman JM, Rajagopal J, Carroll TJ (2008). Canonical wnt signaling regulates Organ-Specific Assembly and differentiation of CNS vasculature. Science.

[19] Wang Y, Rattner A, Zhou Y (2012). Norrin/Frizzled4 signaling in Retinal Vascular Development and Blood Brain Barrier plasticity. Cell.

[20] Zhou Y, Wang Y, Tischfield M (2014). Canonical WNT signaling components in vascular development and barrier formation. J Clin Invest.

[21] Vanhollebeke B, Stone OA, Bostaille N et al Tip cell-specific requirement for an atypical Gpr124- and reck-dependent Wnt/β-catenin pathway during brain angiogenesis. eLife 4:e06489. 10.7554/eLife.06489 10.7554/eLife.06489 PMC4456509 26051822

[22] Qu Q, Sun G, Murai K (2013). Wnt7a regulates multiple steps of neurogenesis. Mol Cell Biol.

[23] Paolinelli R, Corada M, Ferrarini L (2013). Wnt activation of immortalized brain endothelial cells as a Tool for Generating a standardized model of the blood brain barrier in Vitro. PLoS ONE.

[24] Watanabe T, Dohgu S, Takata F (2013). Paracellular barrier and tight Junction protein expression in the immortalized brain endothelial cell lines bEND.3, bEND.5 and mouse brain endothelial cell 4. Biol Pharm Bull.

[25] Gastfriend BD, Nishihara H, Canfield SG (2021). Wnt signaling mediates acquisition of blood–brain barrier properties in naïve endothelium derived from human pluripotent stem cells. eLife.

[26] Morini MF, Giampietro C, Corada M (2018). VE-Cadherin–mediated epigenetic regulation of endothelial gene expression. Circ Res.

[27] Taddei A, Giampietro C, Conti A (2008). Endothelial adherens junctions control tight junctions by VE-cadherin-mediated upregulation of claudin-5. Nat Cell Biol.

[28] Ashburner M, Ball CA, Blake JA (2000). Gene Ontology: tool for the unification of biology. Nat Genet.

[29] Khatri P, Sirota M, Butte AJ (2012). Ten years of pathway analysis: current approaches and Outstanding challenges. PLoS Comput Biol.

[30] Maere S, Heymans K, Kuiper M (2005). BiNGO: a Cytoscape plugin to assess overrepresentation of Gene Ontology categories in Biological Networks. Bioinformatics.

[31] Draghici S, Khatri P, Bhavsar P (2003). Onto-Tools, the toolkit of the modern biologist: Onto-Express, Onto-Compare, onto-design and onto-translate. Nucleic Acids Res.

[32] Subramanian A, Tamayo P, Mootha VK (2005). Gene set enrichment analysis: a knowledge-based approach for interpreting genome-wide expression profiles. Proc Natl Acad Sci U S A.

[33] Dennis G, Sherman BT, Hosack DA (2003). DAVID: database for annotation, visualization, and Integrated Discovery. Genome Biol.

[34] Mi H, Ebert D, Muruganujan A (2021). PANTHER version 16: a revised family classification, tree-based classification tool, enhancer regions and extensive API. Nucleic Acids Res.

[35] Montesano R, Pepper MS, Möhle-Steinlein U (1990). Increased proteolytic activity is responsible for the aberrant morphogenetic behavior of endothelial cells expressing the middle T oncogene. Cell.

[36] Bentzinger CF, von Maltzahn J, Dumont NA (2014). Wnt7a stimulates myogenic stem cell motility and engraftment resulting in improved muscle strength. J Cell Biol.

[37] Bikkavilli RK, Avasarala S, Van Scoyk M (2015). Wnt7a is a novel inducer of β-catenin-independent tumor-suppressive cellular senescence in Lung cancer. Oncogene.

[38] Huang X, Zhu H, Gao Z (2018). Wnt7a activates canonical wnt signaling, promotes Bladder cancer cell invasion, and is suppressed by miR-370-3p. J Biol Chem.

[39] Carmon KS, Loose DS (2008). Secreted frizzled-related protein 4 regulates two Wnt7a signaling pathways and inhibits proliferation in Endometrial Cancer cells. Mol Cancer Res.

[40] Huang S-MA, Mishina YM, Liu S (2009). Tankyrase inhibition stabilizes axin and antagonizes wnt signalling. Nature.

[41] Laksitorini MD, Yathindranath V, Xiong W (2019). Modulation of Wnt/β-catenin signaling promotes blood-brain barrier phenotype in cultured brain endothelial cells. Sci Rep.

[42] Raudvere U, Kolberg L, Kuzmin I (2019). G:profiler: a web server for functional enrichment analysis and conversions of gene lists (2019 update). Nucleic Acids Res.

[43] Huang DW, Sherman BT, Lempicki RA (2009). Bioinformatics enrichment tools: paths toward the comprehensive functional analysis of large gene lists. Nucleic Acids Res.

[44] Huang DW, Sherman BT, Lempicki RA (2009). Systematic and integrative analysis of large gene lists using DAVID bioinformatics resources. Nat Protoc.

[45] Shannon P, Markiel A, Ozier O (2003). Cytoscape: a Software Environment for Integrated Models of Biomolecular Interaction Networks. Genome Res.

[46] Palazzo L, Ahel I (2018). PARPs in genome stability and signal transduction: implications for cancer therapy. Biochem Soc Trans.

[47] Jho E, Zhang T, Domon C (2002). Wnt/β-Catenin/Tcf signaling induces the transcription of Axin2, a negative Regulator of the signaling pathway. Mol Cell Biol.

[48] Vigh JP, Kincses A, Ozgür B (2021). Transendothelial Electrical Resistance Measurement across the blood–brain barrier: a critical review of methods. Micromachines.

[49] Liang Y, Li X-Y, Rebar EJ (2002). Activation of vascular endothelial growth factor a transcription in tumorigenic glioblastoma cell lines by an enhancer with cell type-specific DNase I accessibility. J Biol Chem.

[50] Yuen TJ, Silbereis JC, Griveau A (2014). Oligodendrocyte-encoded HIF function couples postnatal myelination and white matter angiogenesis. Cell.

[51] Davis EK, Zou Y, Ghosh A (2008). Wnts acting through canonical and noncanonical signaling pathways exert opposite effects on hippocampal synapse formation. Neural Develop.

[52] Acebron SP, Niehrs C (2016). β-Catenin-independent roles of Wnt/LRP6 signaling. Trends Cell Biol.

[53] Cerpa W, Godoy JA, Alfaro I (2008). Wnt-7a modulates the synaptic vesicle cycle and synaptic transmission in hippocampal neurons. J Biol Chem.

[54] Wang L, Geng J, Qu M (2020). Oligodendrocyte precursor cells transplantation protects blood–brain barrier in a mouse model of brain ischemia via Wnt/β-catenin signaling. Cell Death Dis.

[55] Karnati HK, Panigrahi M, Shaik NA (2014). Down regulated expression of Claudin-1 and Claudin-5 and up regulation of B-Catenin: Association with Human Glioma Progression. CNS Neurol Disord Drug Targets.

[56] Cottarelli A, Corada M, Beznoussenko GV (2020). Fgfbp1 promotes blood-brain barrier development by regulating collagen IV deposition and maintaining Wnt/β-catenin signaling. Dev Camb Engl.

[57] Rickman M, Ghim M, Pang K (2023). Disturbed flow increases endothelial inflammation and permeability via a Frizzled-4-β-catenin-dependent pathway. J Cell Sci.

[58] Wang Y, Cho C, Williams J (2018). Interplay of the norrin and Wnt7a/Wnt7b signaling systems in blood–brain barrier and blood–retina barrier development and maintenance. Proc Natl Acad Sci.

[59] Yamamoto H, Komekado H, Kikuchi A (2006). Caveolin is necessary for Wnt-3a-dependent internalization of LRP6 and accumulation of beta-catenin. Dev Cell.

[60] Liu C-C, Kanekiyo T, Roth B, Bu G (2014). Tyrosine-based Signal mediates LRP6 receptor endocytosis and desensitization of Wnt/β-Catenin Pathway Signaling. J Biol Chem.

[61] Okumura N, Nakamura T, Kay EP (2014). R-spondin1 regulates cell proliferation of corneal endothelial cells via the Wnt3a/β-Catenin pathway. Invest Ophthalmol Vis Sci.

[62] Krock BL, Skuli N, Simon MC (2011). Hypoxia-Induced Angiogenesis. Genes Cancer.

[63] Boso D, Rampazzo E, Zanon C (2019). HIF-1α/Wnt signaling-dependent control of gene transcription regulates neuronal differentiation of glioblastoma stem cells. Theranostics.

[64] Shen X, Li M, Wang C (2022). Hypoxia is fine-tuned by Hif-1α and regulates mesendoderm differentiation through the Wnt/β-Catenin pathway. BMC Biol.

[65] Mazumdar J, O’Brien WT, Johnson RS (2010). O2 regulates stem cells through Wnt/β-catenin signalling. Nat Cell Biol.

[66] Kaidi A, Williams AC, Paraskeva C (2007). Interaction between β-catenin and HIF-1 promotes cellular adaptation to hypoxia. Nat Cell Biol.

