# Supplementary material for: Wnt7a Decreases Brain Endothelial Barrier Function Via β-Catenin Activation
Source: Mol Neurobiol. 2023 Dec 26;61(7):4854–67. doi: 10.1007/s12035-023-03872-0 (PMC11236883; doi:10.1007/s12035-023-03872-0)
Supplement: Supplementary file 1 — Supplementary Material 1 [file 12035_2023_3872_MOESM1_ESM.pdf]

Supplementary Material

Wnt7a decreases brain endothelial barrier function via β-catenin activation

Molecular Neurobiology

Narek Manukjan[1,2,3], Steven Chau[1], Florian Caiment[4], Marcel van Herwijnen[4], Hubert J. Smeets[4,5], Daniel Fulton[3], Zubair Ahmed[3,6,†], W. Matthijs Blankesteijn[1,2,†], Sebastien Foulquier[1,2,5,7,†]

[†] Co-corresponding author

Author affiliations:

1. *Department of Pharmacology and Toxicology, Maastricht University, P.O. Box 616, 6200 MD Maastricht, The Netherlands*
2. *CARIM - School for Cardiovascular Diseases, Maastricht University, P.O. Box 616, 6200 MD Maastricht, The Netherlands*
3. *Neuroscience and Ophthalmology, Institute of Inflammation and Ageing, University of Birmingham, Edgbaston, Birmingham, B15 2TT, UK*
4. *Department of Toxicogenomics, GROW – School for Oncology and Developmental Biology, Maastricht University, P.O. Box 616, 6200 MD Maastricht, The Netherlands*
5. *MHeNs—School for Mental Health and Neuroscience, Maastricht University, P.O. Box 616, 6200 MD Maastricht, The Netherlands*
6. *Centre for Trauma Sciences Research, University of Birmingham, Edgbaston, Birmingham, B15 2TT, UK*
7. *Department of Neurology, Maastricht University Medical Center+, P.O. Box 5800, 6202 AZ Maastricht, The Netherlands*

Correspondence to: Professor Zubair Ahmed

Full address Neuroscience and Ophthalmology, Institute of Inflammation and Ageing, University of Birmingham, Edgbaston, Birmingham, B15 2TT, UK

E-mail z.ahmed.1@bham.ac.uk

Correspondence to: Dr W. Matthijs Blankesteijn

Full address Department of Pharmacology and Toxicology, Maastricht University, 50 Universiteitssingel, 6229ER Maastricht, The Netherlands

E-mail wm.blankesteijn@maastrichtuniversity.nl

Correspondence to: Dr Sebastien Foulquier

Full address Department of Pharmacology and Toxicology, Maastricht University, 50 Universiteitssingel, 6229 ER Maastricht, The Netherlands

E-mail s.foulquier@maastrichtuniversity.nl

Supplementary Table 1: Primary and secondary antibodies used for immunocytochemistry.

Primary antibody	Host	Dilution	Cat no.	Supplier
Anti-Active β-catenin	Mouse	1:300	05-665	Millipore
Anti-Claudin-5	Mouse	1:300	35-2500	Invitrogen
Anti-Occludin	Rabbit	1:300	40-6100	Invitrogen
**Secondary antibody**				
Anti-Mouse IgG Alexa Fluor 488	Donkey	1:200	A-21202	Invitrogen
Anti-Rabbit IgG Alexa Fluor 594	Donkey	1:200	A-21207	Invitrogen

Supplementary Table 2: Primers used in quantitative PCR.

Primer name	Primer sequence	
Rpl13a	Forward	AGCCTACCAGAAAGTTTGCTTAC
	Reverse	GCTTCTTCTTCCGATAGTGCATC
Ywhaz	Forward	GAAAAGTTCTTGATCCCCAATGC
	Reverse	TGTGACTGGTCCACAATTCCTT
Gusb	Forward	CCGACCTCTCGAACAACCG
	Reverse	GCTTCCCGTTCATACCACACC
Axin2	Forward	AACCTATGCCCGTTTCCTCTA
	Reverse	GAGTGTAAAGACTTGGTCCACC
Cldn5	Forward	CCACGGCCAATGGCGATTAC
	Reverse	TCGTCATCCACACACGGCTT
Ocln	Forward	CCTCGGTACAGCAGCAATGG
	Reverse	TAGTGGTCAGGGTCCGTCCT
Hif1α	Forward	AATGAAGTGCACCCTAACAAGCCG
	Reverse	TGGCCCGTGCAGTGAAGC
Vegfa	Forward	GCACATAGAGAGAATGAGCTTCC
	Reverse	CTCCGCTCTGAACAAGGCT
